# ‘We’re all doing different things’ — exploring primary care practitioners' perspectives of managing distress: a qualitative study

**DOI:** 10.3399/BJGP.2024.0820

**Published:** 2025-10-07

**Authors:** Hannah Bowers, Carolyn A Chew-Graham, Miriam Santer, Harm Van Marwijk, Berend Terluin, Tony Kendrick, Paul Little, Michael Moore, Manoj Mistry, Debs Smith, Al Richards, Bronwyn Evans, Nikki Lester, Roya Kolahy, Adam W A Geraghty

**Affiliations:** 1 School of Primary Care, Population Sciences and Medical Education, University of Southampton, Southampton, UK; 2 School of Medicine, Keele University, Keele, UK; 3 Midlands Partnership NHS Foundation Trust, Stafford, UK; 4 Department of Primary Care and Public Health, Brighton and Sussex Medical School, Brighton, UK; 5 Department of General Practice, Amsterdam University Medical Centre, Amsterdam, the Netherlands

**Keywords:** distress, general practice, mental health, anxiety, depression, qualitative research

## Abstract

**Background:**

Distinguishing emotional distress from mental health problems such as anxiety and depression can be difficult for clinicians. Both commonly present and are managed in primary care. There are likely to be important differences in the management of emotional distress compared with anxiety and/or depression, but the current nature of assessment and management is unclear.

**Aim:**

To explore how emotional distress is understood and how people are managed by a range of practitioners in primary care settings in the UK.

**Design and setting:**

A qualitative study using semi-structured interviews with primary care practitioners in the UK.

**Method:**

Online interviews were conducted with practitioners who directly assess patients with mental health symptoms, including GPs, nurse practitioners, social prescribers, and mental health practitioners. Recruitment was via a digital poster circulated by research delivery networks. Interviews covered how practitioners understood and identified distress, the support provided to patients, and challenges assessing and managing emotional distress. Verbatim transcriptions were analysed using an inductive thematic approach.

**Results:**

In total, 29 interviews were conducted and four themes were developed in collaboration with the wider team, including public contributors: the multifaceted nature of distress; ‘We’re all doing very different things’; managing and understanding distress is challenging; and demedicalising distress in the face of increasing societal pressures. Complexity was driven by the wide-ranging professionals involved, complex patient circumstances, systemic challenges, and societal contexts.

**Conclusion:**

Identifying and managing emotional distress is complex, variable, and challenging. Complexity appears to be increasing through the systemic challenges and range of professionals involved.

## How this fits in

Previous research with patients and GPs has highlighted that ‘emotional distress’ and anxiety and/or depression are commonly seen in primary care and are difficult to distinguish. In this study, we illustrate how distress is conceptualised and managed by a broad range of practitioners in primary care, including those in newer roles introduced as part of the Additional Roles Reimbursement Scheme. Our findings highlight significant challenges, complexity, and variability in both identifying distress and supporting patients experiencing distress. These challenges were seen to be driven by current contextual factors such as overmedicalisation of mental health, increasing social pressures, and systemic challenges within primary care settings.

## Introduction

GPs estimate around 40% of their consultations involve mental health.^1^ They are responsible for detecting and treating ‘common mental health problems’.^2,3^ Ninety per cent of people with mental health problems are being supported entirely within primary care.^4^

Common mental health problems such as depression and anxiety are defined in diagnostic manuals with guidelines to inform management.^5,6^ However, there is long-standing criticism of a disease-model approach to depression, which is focused on diagnostic criteria and symptom duration and severity.^7^ There is debate about the distinction between a depressive and/or anxiety disorder and a (potentially severe) emotional response with functional impairment that may not represent psychopathology. The latter has been conceptualised as ‘distress’^8,9^ and is very common in primary care.^10,11^

There are evidence-based guidelines for the management of depressive and anxiety disorders,^5^ whereas there are no such guidelines for supporting people experiencing distress. Clinicians and patients may agree that their symptoms do not reflect psychopathology, rather they may be driven primarily by difficult life circumstance.^12,13^ Nonetheless, diagnostic labels may be used in order to enable access to some form of treatment (such as antidepressant medication), where options for those experiencing distress might be limited.^14^

Thus, this is an area where clinicians face complexities regarding appropriate identification^13,15^ and related complexities regarding appropriate management.^16^ Further compounding factors include increasing levels of distress nationally following the COVID-19 pandemic and the cost of living crisis, alongside rising antidepressant prescriptions, and urgent calls to address this rise.^17–19^

Patients experiencing the full range of symptoms including depression and/or anxiety and distress are now managed by a varied and growing number of practitioners in primary care. NHS England’s introduction and roll-out of the Additional Roles Reimbursement Scheme (ARRS) in 2019 expanded the recruitment of a wide range of practitioners including clinical pharmacists, mental health practitioners, and social prescribing link workers.^20^ These practitioners play key roles in supporting mental health, including both identification and management. It is critical to understand how the increasingly broad array of practitioners understand and work in this area to ensure appropriate and effective care is provided.

Our aim in this qualitative study was to explore how distress (as opposed to mental illness) is understood and managed by a range of practitioners in primary care settings in the UK.

## Method

### Study design

We conducted a qualitative study using semi-structured interviews to explore UK primary care practitioners' perceptions and experiences regarding the management of people with distress.

### Participants

Three research delivery networks shared information about the study with primary care practices. Practices then shared this with staff via email. Practitioners were also recruited via team networks and snowball sampling.

Primary care practitioners who directly assess patients with mental health symptoms were eligible to take part. Practitioners were purposively sampled to ensure diverse recruitment with regards to gender, ethnicity, age, role, and years’ experience.

### Data collection

Semi-structured interviews (up to 60 min) took place via video call or telephone (using Microsoft Teams). Interviews were conducted by the first author, a female senior research fellow with a PhD in psychology who co-led the project with the senior author. Interviews took place between November 2023 and April 2024. The interviewer had no relationship with participants prior to interviews. Interviews were audio-recorded and transcribed verbatim. The interview schedule (see Supplementary Box S1) explored how practitioners conceptualise distress compared with depression and anxiety and their approach to management.

### Data analysis

Data were analysed thematically, informed by the steps of reflexive thematic analysis,^21^ combined with a ‘codebook approach’ that facilitates collaboration in applied research.^22,23^ Familiarisation and coding were completed by the first author using NVivo (version 14). Data extracts were discussed at regular team meetings. This informed iterative development of codes and themes. The team comprised behavioural scientists, academic GPs, a mental health nurse, and three public contributors. Information power was considered during discussions and recruitment finished when the team agreed there was sufficient information power.^24^

### Public involvement

A diverse group of public contributors with lived experience of distress and/or depression participated in regular team meetings to discuss the data, coding, and theme development. They informed the study aims and the interview schedule, and contributed to this article.

## Results

### Participants

In total, 29 participants were recruited from 74 expressions of interest across 36 practices (see Table 1). The mean age was 42.6 years (range 22–69) and they had been in their role for 5.8 years on average (range 5 months to 24 years).

**Table 1.** Demographic characteristics of the recruited sample (*N* = 29)

Characteristic	*n*
**Role**	
GP	7
Nurse practitioner	4
Mental health practitioner (including mental health practitioners, advisors, and coaches)	5
Social prescriber	5
Mental health occupational therapist	4
Paramedic	1
Pharmacist	1
Mental health nurse	1
Advanced nurse practitioner	1
**Gender**	
Man	7
Woman	22
**Ethnicity**	
Asian or Asian British	4
Black, Black British, Caribbean or African	2
White British	21
Other White	2
**Interest, experience, or training in mental health**	
None	7
Some interest, experience, and/or training	22

### Thematic analysis

**Figure 1.**
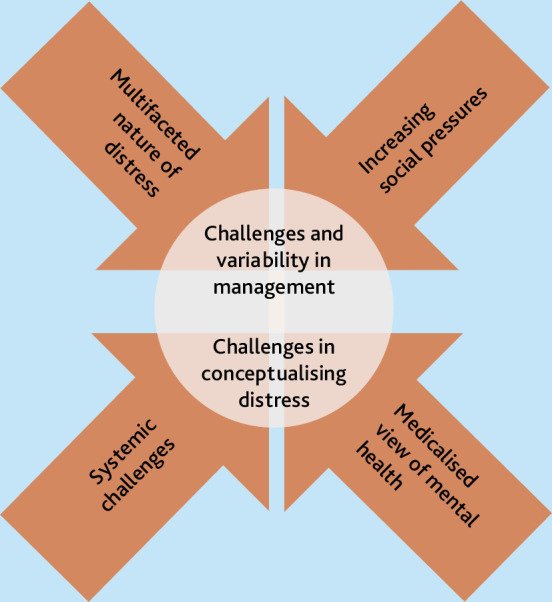
Diagram of thematic findings.

Four themes (see Supplementary Box S2 for example codes) were developed that encapsulated how distress was conceptualised and managed in primary care: the multifaceted nature of distress, ‘We’re all doing very different things’, managing and understanding distress is challenging, and demedicalising distress in the face of increasing societal pressures. Figure 1 illustrates the findings.

### The multifaceted nature of distress

Distress was considered to be highly complex and multifaceted. Wide-ranging contributing factors were highlighted (such as bereavement, finances, housing, employment, health conditions, neurodivergence, and abuse). Participants emphasised a need for individualised support to suit patients’ complex circumstances:

*'It’s very person-centred in terms of it’s not just, okay, you're distressed, so this is what we do with distressed people. It’s very personalised to their exact issues at that time*.*'* (Mental health occupational therapist [MHOT]07001)

This complexity was compounded by cultural factors (such as some cultures being less likely to speak to non-medical practitioners), language (such as difficulties using interpreters), and refugee status (particularly regarding isolation in rural areas). Relative social deprivation was reported to be a common characteristic of patients experiencing distress. For some participants this created additional challenges regarding the patient’s ability to manage their distress, their ability to make lifestyle changes (for example, time off work or changing jobs), and their use of drugs and alcohol:

*'They're people that generally speaking would be the poorer members of society who just don't have the agency, the networks, the social networks to be able to change what’s going on for them at the moment. I'd say almost all of them have experienced childhood trauma, and almost all of them experiencing pain and some physical health problems alongside everything else, lots of housing issues and often experiences of loss and bereavement*.*'* (Social prescriber [SP]09001)

### ‘We’re all doing very different things’

There were a wide range of practitioners involved in supporting people experiencing distress. They were providing different support because of a need for individualised care, varied skillsets, and uncertainty around novel roles. They often worked collaboratively drawing on their specific skill set and expertise:

*'I feel like it’s so new in our area. We're all just doing our own thing and fumbling our way through … I feel like we're all doing very different things*.*'* (MHOT10005)

Support largely fell into five broad categories: supportive listening, practical support (such as problem-solving and supporting lifestyle changes), signposting and referring, techniques for managing distress, and medication.

Supportive listening was considered an important way to show empathy, validate and normalise emotions, and offer reassurance by a range of practitioners. Normalisation also served to counter patients’ medicalised view of distress:

*'I think, sometimes, people just need to talk to the GP; and* [for] *some, the doctor is medicine. I'm sure that sometimes helps — and just being able to tell people that it’s normal to feel awful, when someone’s just died — that sort of thing. Try not to medicalise the symptoms*.*'* (GP09001)

Mental health practitioners and mental health occupational therapists described providing strategies for coping with distress (for example, distress tolerance techniques from dialectical behavioural therapy). They sometimes had workbooks or handouts they provided to patients. Many also addressed lifestyle factors that may exacerbate distress such as alcohol use and sleep hygiene:

*'I think people come in saying, I'm feeling like this, and I shouldn't be feeling like this, and you've got to make this stop. So I think a lot of the work that we try to do is say, no, actually, it’s okay to sit with that distress, but here’s how you can cope with it*.*'* (MHOT10002)

Mental health occupational therapists and mental health practitioners sometimes worked alongside social prescribing link workers who offered more practical support, signposting and empowering patients to help themselves.

Social prescribing link workers recognised their role was not specifically in mental health, but despite this, mental health (particularly distress) was something they see regularly. Signposting to services to address the specific stressor (such as bereavement support groups or Citizen’s Advice) was discussed by the vast majority of participants. There was variability in services known to be offered that were dependent on local availability. Social prescribing link workers were seen by participants as having expertise in signposting to community support, and many of the participants said that they referred patients to social prescribing link workers:

*'*O*ur role as social prescribers is to give people time … Although they may have mental health problems, ultimately there are many other things that would also be affecting them ... So we obviously try and listen to them, we try and hear what they're saying, and then we try and link them up with services that will help them with those worries*.*'* (SP06004)

Many participants talked about signposting patients to NHS Talking Therapies for support – however one practitioner with experience assessing patients for NHS Talking Therapies recognised that this is not always appropriate for people experiencing distress caused by an external stressor:

*'It’s hard to then say, "Oh, try to be more motivated", because they are motivated to try and get themselves better, but they just weren't getting the support from other services, like the council, for example, so it didn't necessarily fit with what our therapy could do*.*'* (Mental health practitioner [MHP]13002)

Participants shared contrasting views about medication. Some participants described prescribing antidepressants for patients experiencing distress if that distress was having an impact on their ability to function, if it was of a longer duration, or getting worse. Many of the interviewed GPs reported avoiding prescribing antidepressants for distress in favour of NHS Talking Therapies or social prescribing. Some non-GP participants reported working with GPs who often prescribe antidepressants for people experiencing distress. These participants felt GPs’ time-limited consultations make exploration of patients’ circumstances difficult. They felt this increased the likelihood of a depression diagnosis and antidepressant prescription:

*'They're on antidepressants and things like that, and again, they'll be on 50 milligrams or whatever, and then it’s like, it’s not working so I'm going to go back and speak to my GP about upping it, and it goes up, and then it changes medication, and then it goes up. It never seems to be coming down … You speak to people, and they've not actually had a conversation with the GP. The GP doesn't know that relationship problems have happened or something’s happening at work*.*'* (MHP10007)

Some participants also talked about short-term medication use to manage distress (such as benzodiazepines, beta blockers, and medication to improve sleep).

### Managing and understanding distress is challenging

Participants recognised that it can be difficult to differentiate between distress and depression and anxiety. GP participants described differences more readily than other practitioners who took longer to reflect before responding. Ultimately, participants were largely in agreement about what the differences are: distress has an external cause that is often time limited, whereas depression and anxiety have other characteristic symptoms and may have a greater impact on functioning.

Most practitioners felt it was useful to distinguish between distress and disorder. For GPs (who described their role as diagnosing and prescribing) the distinction was important for determining treatment, particularly medication:

*'I'm sure it must be useful to work out whether they're suffering with depression or anxiety, or some other mental health issue, because that I'm sure would influence how you manage them. It could add to how you manage them. In terms of managing the distress, if they're distressed because of, they can't feed their family that week, but they're otherwise not normally suffering with mental health problems, then we might have ways to access food banks and get support from the social prescribers, things like that. So there might be lots of things there, but if they also suffered with depression, then you might also look at talking therapies, or medication, or other things as well*.*'* (GP09001)

Some practitioners (particularly mental health occupational therapists, mental health practitioners, and social prescribing link workers) felt that the label of distress, depression, and/or anxiety was less relevant and instead focused on the patient’s complex individual circumstances to determine management, regardless of diagnostic label:

*'My job is to hear what that person says, the meaning that person gives to everything in their lives and then together we co-create a plan to change, so it doesn't matter if that person has a diagnosis or not. It doesn't change anything*.*'* (SP09001)

Participants frequently highlighted the need to have an in-depth conversation with the patient in order to identify distress and determine management. Short appointments were seen as a barrier to this, particularly for GPs, whose shorter appointments were seen to increase the likelihood of a depression diagnosis:

*'*... *the GP was saying, "I think he’s really low in mood". So I had an appointment with him and that’s where we explored in a bit more depth about all of the changes that had happened because of the loss of the job … the GP had prescribed antidepressants, actually, but he hadn't taken them … So we did a lot of work about, at that point in time, rebuilding that resilience … He didn't pick up the prescription. Said he just felt better*.*'* (MHOT10002)

This distinction was also guided by the patient’s medical records and some participants reported supplementing conversations with screening tools (for example, the Patient Health Questionnaire^25^ [PHQ]) to support decisions about providing a diagnostic label:

*'I mean, we're quite lucky because a lot of our patients come pre-triaged on our eConsult, and so they will have done PHQs and GAD* [Generalised Anxiety Disorder screening tool]*, and all the rest of it. So that will also help sway the decisions, especially if they're scoring really high on a PHQ or really high on a GAD-7. You'd be like, oh gosh, maybe we should be thinking about giving them a diagnosis*.*'* (GP01001)

Some practitioners used questionnaires less frequently or only to evidence a perceived need for medication. One mental health occupational therapist described her reluctance to use questionnaires as they fail to capture the complexity of the patient’s situation:

*'From my experience of working in mental health, I don't find that they* [questionnaires] *often give a true representation of what the patient’s actually going through*.*'* (MHOT10002)

Five practitioners expressed less confidence in managing distress because of perceived limited expertise in mental health:

*'No, I can't say I do feel confident because it’s — again, mental health isn't my field. I'm an adult-trained nurse. So it’s not my comfort zone in any way, shape or form, but I can't turn that individual away so try to manage it in a safe a way as possible, but I'm not confident or comfortable with it on most occasions.'* (Nurse practitioner 08001)

Other practitioners were more confident in their approach, sometimes drawing on their experiences from previous employment outside of primary care.

Participants identified many systemic challenges to managing distress such as long waiting times for services, inappropriate referrals (within primary care), and a lack of service provision:

*'So I think a lot of patients that end up with us should never get as far as us. I think they should go straight to the talking therapies, but there’s such a long wait on that they don't have access. I think they often re-present to us because they're not getting anywhere. I think we prescribe more because we have no other option*.*'* (GP06001)

This resulted in some participants feeling frustrated or like a failure. For some participants managing distress affected their own wellbeing. Some reported experiencing distress and vicarious trauma.

Some participants took a *‘scattergun’* approach to referrals that they felt exacerbated long waiting times:

*'Then you feel a bit guilty because it’s a bit of a scattergun effect to which service will pick you up first, and then you know you're causing a problem because you're bottlenecking all the services, but you just want to get some help as soon as possible for them*.*'* (MHOT10005)

Some practitioners, particularly ARRS practitioners, had guidance about the duration they should see a patient (for example, no longer than 6 months). This contradicted the benefits seen in practice by supporting people for longer periods. Practitioners talked about prioritising the patients’ needs over the guidance from management (often external to the practice):

*'We're managing patients that maybe no one else in the surgery — not hasn't been able to, but it hasn't been appropriate for anyone else. I will carry on and do what is right for the surgery, but then I have guidelines from my employers saying you only have up to six months to see this person*.*'* (SP13001)

### Demedicalising distress in the face of increasing societal pressures

Participants highlighted the changing context of mental health as a challenge in managing distress. Society’s destigmatisation of common mental health problems was viewed as creating a medicalised view of distress:

*'In my view, there’s been a lot of emphasis on destigmatising mental health … they come in and say, I'm depressed … I find a lot of what I end up doing is talking to them about actually, it’s not a depression, it’s not a mental illness. What you're experiencing is a really normal reaction to a life event*.*'* (MHOT10002)

This medical view was related to challenges in supporting patients owing to patient expectations of a *‘quick fix’* or medication to treat their distress. This had an impact on the patient’s motivation and sense of responsibility (where some patients felt it was the clinician’s responsibility to treat the distress):

*'Some of them are disappointed that I don't immediately offer them medication — maybe they want a quick fix*.*'* (GP09001)

This was compounded by reports of increasing distress, particularly in relation to the current cost of living crisis. Some practitioners suggested that people have less tolerance for distress in this context:

*'I feel there’s a lot less ability to cope now and I think partly that’s because of the pressures of life, that most people are so stressed that there’s no room for dealing with anything else*.*'* (GP12001)

Short appointment length and long waiting times for psychological therapy were seen to contribute to medicalising distress through increasing antidepressant prescriptions. One social prescribing link worker described how demedicalising distress could relieve some of the pressure on services by directing people away from GPs towards support from other practitioners and the community:

*'*[social prescribing] *is an empowering strengths-based model for people that increases confidence, and that is what we need if we're not going to burden the NHS with endless things. If we have a paternalistic view of things where we feel something is wrong with us that we can't possibly change, we need a doctor … That’s just going to increase the burden on the NHS*.*'* (SP09001)

## Discussion

### Summary

These findings highlight the variability in managing distress in primary care. Variability in management was seen as crucial to address the wide-ranging patient circumstances, which often intersected with deprivation.

Distinguishing between distress and disorder was challenging and a demedicalised view of distress was favoured by many participants. Practitioners supported patients by normalising emotions and offering support for the external stressor, often provided by non-medical practitioners. Some of these practitioners also supported patients to self-manage the feelings of distress. Practitioners’ short appointment times and a paucity of timely and appropriate support impeded management.

### Strengths and limitations

This study recruited a wide range of practitioners in primary care, including those from newer ARRS roles. This has enabled the exploration of distress management from a wider and more novel perspective in primary care. Recruiting and interviewing many practitioners without a specific interest in mental health allowed for exploration of a broad range of practitioners’ views, highlighting that many lack confidence in supporting people with mental health difficulties.

Participants were recruited from three CRNs in England. Although many practices covered deprived regions, allowing for consideration of how deprivation has an impact on distress, there is likely additional variability across other regions. The gender split of participants across the roles was somewhat reflective of the workforce. Although we deliberately sought views of men in roles predominantly held by women, recruitment of more women GPs may have enhanced the data. The ethnicity of participants is approximately reflective of the population; however, many of the practices recruited in this study had predominantly white patient populations. Further research should explore the impact of ethnicity on the presentation and management of distress, particularly as our findings suggest some groups may seek support from particular practitioners over others.

### Comparison with existing literature

The predominant understanding of distress was that it was caused by an external stressor, but some felt that distress was a milder precursor to depression. These views, reported by a broad range of practitioners, are similar to those of GPs reported in a previous qualitative study.^13^ Some practitioners used screening tools to aid decision-making about depression diagnosis and prescribing antidepressants. However, in a primary care cross-sectional study, almost half of the participants who scored as possibly depressed using the PHQ-9 were categorised as distressed but not depressed by the Four-Dimensional Symptom Questionnaire^10^ (4DSQ, measuring distress, depression, anxiety, and somatisation as separate constructs).^26^ Practitioners’ use of the PHQ-9 with patients experiencing distress may therefore result in overdiagnosis of depression and overprescription of antidepressants.

Some GPs described how distress is better managed by non-medical professionals in primary care to address the social causes of distress. Support was frequently signposted by a social prescribing link worker and required significant tailoring to address the wide-ranging causes of distress, often (but not always) related to deprivation. A systematic review of uncontrolled evaluations demonstrated that social prescribing for mental health can improve outcomes, reduce GP consultations, and reduce prescriptions.^27^ Although practitioners have previously acknowledged there is a greater need for social prescribing in areas of deprivation,^28^ there are accessibility barriers (such as affordability and transport issues).^29^ This may be addressed through coproduction to improve accessiblity.^30^ Practitioners mentioned signposting to community and voluntary sector services, but discussions of this issue in the data were limited. Further qualitative work should explore the patients’ and practitioners’ experiences of these services in supporting people experiencing distress.

Some practices had mental health occupational therapists or mental health practitioners who conducted psychotherapeutic work to help patients self-manage distress. However, there are concerns about shortages of these practitioners.^31^

To counter the disease-model of depression in UK primary care, Dowrick proposed a shift from reporting symptom severity to instead exploring the physical, psychological, and social circumstances of the patient, which does not require a diagnostic label.^32^ In the current study, distinguishing between distress and depression was considered useful to determine management, particularly where medication is concerned. However, some practitioners (particularly social prescribing link workers and mental health occupational therapists) argued that the label is less important and instead focused on providing empathy, empowerment, and individualised support for people experiencing distress.

Short consultations were reported to make it challenging to distinguish between distress and depression and/or anxiety, which may sometimes result in prescription of antidepressants (particularly for GPs). Most practitioners felt that antidepressants were useful for depression and not distress. However, previous qualitative work with GPs demonstrates how antidepressants are sometimes offered (particularly in areas of deprivation) because of limited access to alternative interventions.^14^ In the current study, longer appointments were said to facilitate better understanding of distress, thereby directing people to appropriate care; however, those responsible for diagnosing and prescribing (that is, GPs) had the shortest appointment times.

Practitioners reported that patients often experienced distress with complex multimorbidity as well as challenging social circumstances. Multimorbidity may complicate consultations through increased appointment length to cover multiple topics and further difficulty identifying distress within a complex presentation of multiple psychological and physical symptoms.^33^ Many participants viewed distress as non-medical and highlighted a need to demedicalise distress to reduce the pressures in primary care and better support people experiencing distress. This may be achieved through normalising patients’ experiences, reducing prescriptions for antidepressants, and increasing provision of psychological and social support provided by ARRS roles. Further research is needed to explore patients’ experiences of these new roles in primary care and their impact on patient outcomes.

### Implications for research and practice

Current pressures make it difficult to help people manage their distress in primary care. There is a need to improve how patients are directed to services within and outside of primary care to reduce inappropriate referrals and waiting times. This is increasingly vital as broader contexts continue that are likely to increase distress (such as the cost of living crisis, geopolitical instability, and the climate crisis).^18,19,34,35^ This may be achieved through training practitioners in identifying and supporting distress (for example, through improved social prescribing).^28^ Research should develop processes that facilitate more rapid delineation between distress and disorder, particularly for GPs. For example, this could include exploring the utility and feasibility of tools that may support this distinction (such as the 4DSQ), supporting consultation approaches that more readily allow for this distinction, and/or increasing consultation length.^36^ This may reduce antidepressant prescribing, normalise patients’ experiences, and direct patients to more suitable support. This demedicalised approach to distress would direct patients to support that addresses the (often social) causes of distress, which may alleviate some of the pressures in primary care, particularly for GPs.

Mental health occupational therapists and mental health practitioners appear to play an essential role in helping patients to self-manage distress, while social prescribing link workers vitally support patients to address the external stressor. Expansion of these roles may benefit patients and practices but may not be feasible because of workforce shortages. Other methods to support patients to self-manage symptoms of distress, such as guided online support or workbooks, may therefore prove useful.

The conceptualisation and management of distress in primary care is complex and highly variable. Improvements are needed to support practitioners to identify and manage people experiencing distress to address systemic challenges, overmedicalisation, and increasing societal pressures driving distress.
